# Oncolytic viral therapy for nonmelanoma skin cancer and cutaneous lymphoma – A systematic review

**DOI:** 10.1016/j.jdin.2024.11.010

**Published:** 2025-02-07

**Authors:** Felicia Li Ling Ong, Darryl Kai Xian Chin, Yiming Zhu, Choon Chiat Oh

**Affiliations:** a Department of Dermatology, Singapore General Hospital, Singapore, Singapore; b Yong Loo Lin School of Medicine, National University of Singapore, Singapore, Singapore; c Duke-NUS Medical School, Singapore, Singapore

**Keywords:** epidemiology, nonmelanoma skin cancer, oncolytic virus, virotherapy

## Abstract

The rising incidence and consequent health care burden of nonmelanoma skin cancers, including cutaneous lymphomas, are a growing cause for concern. Oncolytic viruses (OVs) are emerging immunotherapies with limited literature on their use. We conducted a systematic review to evaluate their role in nonmelanoma skin cancer and cutaneous lymphoma treatment (CRD42024526854). We identified 11 published studies involving a total of 20 patients (squamous cell carcinoma *n* = 3, Merkel cell carcinoma *n* = 7, cutaneous T cell lymphoma *n* = 9, basal cell carcinoma *n* = 1). OVs used include Talimogene laherparepvec (73%, *n* = 8), measles virus (9%, *n* = 1), vesicular stomatitis virus (9%, *n* = 1), and adenovirus (9%, *n* = 1). Complete response occurred in 67% (*n* = 2) of squamous cell carcinoma cases, 85% (*n* = 6) of Merkel cell carcinoma cases, and 11% (*n* = 1) of cutaneous T cell lymphoma cases. The most common adverse event was fever or flu-like symptoms (*n* = 5, 25%). Fourteen unpublished clinical trials investigating regimes such as OV monotherapy (43%, *n* = 6), combination therapy with existing immunotherapy (21%, *n* = 3), and comparing OV combination versus monotherapy (29%, *n* = 4) or versus immune checkpoint inhibitor alone (7%, *n* = 1). Overall, heterogeneity of existing studies significantly limits generalizability of results. Further research is needed to reveal the potential role of OVs in the future of nonmelanoma skin cancer and cutaneous lymphoma treatment.

**Capsule Summary**

• This article summarizes the current available evidence on oncolytic virotherapy in the treatment of nonmelanoma skin cancers and cutaneous lymphomas.
• Our study highlights the promising evidence, as well as the challenges surrounding oncolytic virotherapy and the need for more robust evidence and research in this area.

## Introduction

Nonmelanoma skin cancers (NMSCs) are among the most common malignancies globally.^1^ While majority are squamous cell carcinomas (SCCs) and basal cell carcinomas (BCCs), and less commonly, Merkel cell carcinomas (MCCs), there also exists several other less common types of skin cancers, such as Kaposi sarcomas, angiosarcomas, and cutaneous lymphomas (CL).^2^ As the overall incidence of skin cancers continues to rise globally, the burden of disease and associated mortality is a growing cause for concern,^2^, ^3^, ^4^ especially for metastatic or relapsed cases which often carry poor prognoses.^5^ This highlights the pressing need for newer and more effective therapies to overcome the limitations of existing systemic treatments.

Amidst the ongoing research on novel therapies for NMSC treatment, oncolytic viruses (OVs) have shown immense promise due to their ability to selectively attack and destroy tumor cells and stimulate antitumor immunity.^6^ Thus far, while several OVs have already been approved globally for treating various malignancies, many more are currently being developed in clinical trials.^6^ Yet, despite the established literature on OVs as an emerging cancer therapy, little is known about its use in treating NMSCs. We conducted a systematic review to assess the role of oncolytic viruses in the treatment of NMSCs, including CLs.

## Methods

Our study sought to determine the utility of oncolytic virotherapy, as compared to standard treatments, in patients with NMSC or CL. The study was registered with PROSPERO (CRD42024526854).

**Fig 1:**
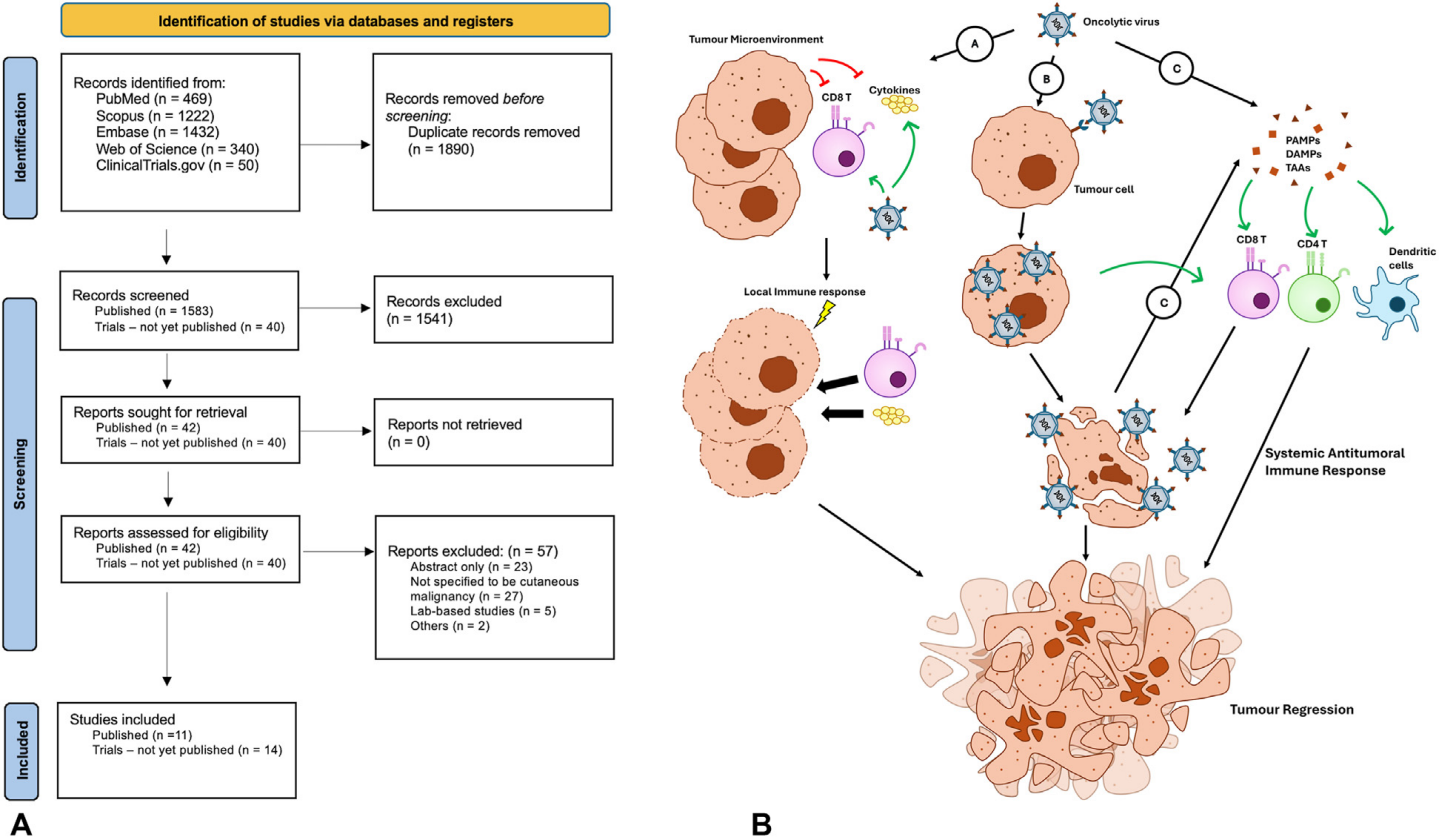
**A,** Summary of systematic review performed according to the Preferred Reporting Items for Systematic Reviews and Meta-Analyses (PRISMA) guidelines. **B,** Mechanisms of Action of Oncolytic Viruses. (A) Remodeling of tumor microenvironment: Viral particles released by oncolytic viruses alter cytokines and immune cells within the tumor microenvironment to stimulate a local immune response. This results in lysis of tumor cells, which at the same time releases neo-antigens that can subsequently trigger a de novo immune response at distant sites. (B) Direct tumor lysis: Direct infection and replication within cancer cells which eventually results in lysis of infected cells. (C) Induction of systemic antitumor immune response: Release of viral pathogen-associated molecular patterns, danger-associated molecular pattern signals, and tumor associated antigens stimulate adaptive immune cells, which then travel to sites of tumor growth and mediate antitumor immunity.

In accordance with the Preferred Reporting Items for Systematic Reviews and Meta-analyses (PRISMA) guidelines, PubMed, Scopus, Embase, Web of Science, and ClinicalTrials.gov databases were searched from inception to 30 March 2024 (Fig 1, *A*). Our search strategy included the use of search terms combining “oncolytic” or “virotherapy” with each skin neoplasm term of “squamous cell carcinoma,” “basal cell carcinoma,” “Merkel cell carcinoma,” “angiosarcoma,” “Kaposi sarcoma,” “extramammary Paget disease” (EMPD), and “lymphoma.” Results were collated and screened after accounting for duplicates. Observational or interventional studies were included if they were published in English and involved the use of OV on human subjects with a histological diagnosis of SCC, BCC, MCC, angiosarcoma, Kaposi sarcoma, EMPD, or lymphoma. Studies in which the malignancy studied was not specified to be of cutaneous origin, as well as animal models, lab-based studies, commentaries or literature reviews, were excluded.

Three independent reviewers screened the titles and abstracts of search results (FO, DC, YZ), followed by a full-text assessment of potentially relevant articles. Any disagreements were resolved with discussion to reach a consensus. For eligible studies, data extraction was performed using a standardized form (Supplementary Appendix A, available via Mendeley at https://data.mendeley.com/datasets/t2h7j69hkw/1) to collect information on study characteristics, patient demographics, tumor stage and location where applicable, treatment regimens, response rates and adverse effects. The quality of these studies was evaluated with the Joanna Briggs Institute checklist or an adapted Risk of Bias in Nonrandomized Studies – of Interventions (ROBINS-I) tool, depending on study type. (Supplementary Appendix B, available via Mendeley at https://data.mendeley.com/datasets/t2h7j69hkw/1)

While our search returned unpublished research including conference abstracts and presentations, only clinical trials were included in our study as we believe these trials would best reflect the latest progress in research in this area. For ongoing clinical trials deemed suitable for inclusion, information on study design were extracted using a separate standardized form. (Supplementary Appendix C, available via Mendeley at https://data.mendeley.com/datasets/t2h7j69hkw/1)

## Results

Our search returned a total of 3513 results. After accounting for duplicates, 1623 were screened to include a total of 11 published studies comprising 8 case reports/case series and 3 clinical trials, involving a total of 20 patients (SCC *n* = 3, MCC *n* = 7, BCC *n* = 1, cutaneous T cell lymphoma (CTCL) *n* = 9) (Tables I and II). No relevant studies on angiosarcoma, Kaposi sarcoma, or EMPD, were found. References for all included studies can be found in Supplementary Appendix D, available via Mendeley at https://data.mendeley.com/datasets/t2h7j69hkw/1.

**Table I:** Published studies on the use of oncolytic virotherapy in nonmelanoma skin cancer treatment

Study	Study type	Number of patients	Diagnosis/location	Tumor stage/metastasis	Age	Treatment		Virus family	Previous treatment	Response	Adverse effects
Lebhar et al, 2023	Case report	1	SCC/left arm	In-transit metastasis	71	Intralesional T-VEC - 32 cycles every 2 wk over a 15-mo period - Initial concentration of 10^6^ pfu/ml followed by 10^8^ pfu/ml for all subsequent treatments External beam RT (6 fractions of 600 cGy) – to 1 new lesion which did not show early signs of response to T-VEC		HSV	Mohs micrographic surgery, adjuvant external beam RT	No evidence of active disease at 13 mo	Thrombocytopenia – but deemed unlikely related to T-VEC treatment
Nguyen et al, 2021	Case report	1	SCC/scalp	In-transit and lymph node metastasis	85	Intralesional T-VEC - 2 sessions of 10^6^ pfu/mL 1 mo apart		HSV	Surgical excision, adjuvant RT, subsequent Mohs micrographic surgery for recurrence	Regression noted in all clinically visible lesions after second session In complete remission after almost 2 y	Not reported
Jhawar et al,∗ 2023	Case report	1	SCC/post auricular scalp	Lung metastasis	Late 60s	Combination T-VEC, nivolumab and palliative RT (50 Gy in 20 fractions)		HSV	Cemiplimab, surgical excision	Near complete resolution with combination therapy with no evidence of progression at 44 mo	Not reported
Blackmon et al,† 2017	Case series	2	MCC/right cheek	Dermal metastasis	87	Intralesional T-VEC - Initial dose of 2 mL of 10^6^ PFU, followed by 3 doses of 1-2 mL of 10^8^ PFU/mL at 2-wk intervals		HSV	Surgical resection, adjuvant RT	Complete response at 9 wk form treatment initiation and at 5 mo from last dose	Mild fatigue
			MCC/scalp	Lymph node metastasis	77	Intralesional T-VEC - Initial dose of 1 mL of 10^6^ PFU, followed by 7 doses of 1-2 mL of 10^8^ PFU/mL at 2-wk intervals			Surgical resection	Partial response persisting at 7 mo from last T-VEC dose	Mild fatigue, nausea, injection site tenderness
Westbrook et al, 2019	Case series	4	MCC/right cheek	Dermal metastasis	87	Intralesional T-VEC - Initial dose of 1-4 mL of 10^6^ pfu/mL - 3 wk later, given subsequent doses of 1-4 mL of 10^8^ pfu/mL at 2-wk intervals	Total 4 doses	HSV	Surgical resection, adjuvant RT	Complete response, no recurrence at >24 mo	Not reported
			MCC/scalp	Lymph node metastasis	77		Total 8 + 7 doses		Surgical resection	Complete response, no recurrence at >24 mo	Mild fatigue, transient nausea
			MCC/left cheek	Parotid and post auricular nodal metastasis	81		Total 8 doses		Surgical resection	Complete response, no recurrence at >10 mo	Mild transient flu-like symptoms
			MCC/right anterior neck	Dermal metastasis	76		Total 5 doses		Surgical resection	Complete response, no recurrence at >13 mo	Not reported
Nguyen et al, 2019	Case report	1	MCC/buttock	In-transit metastasis in left thigh, lower back and left calf	66	Intralesional T-VEC - Initial dose of 2 mL of 10^6^ pfu/mL, followed by 4 mL of 10^8^ pfu/mL at 2-wk intervals		HSV	Surgical resection, adjuvant RT, adjuvant cisplatin/etoposide, pembrolizumab	Complete response at 8 mo, no recurrence at 2 y	Fever, chills, fatigue
Cilento et al, 2022	Case report	1	MCC/left hand	Multiple in transit metastases	75	Intralesional T-VEC (exact regimen not reported; total treatment duration 6 mo)		HSV	Surgical resection, adjuvant RT, avelumab, peptide receptor radionuclide therapy, carboplatin	Near complete response at 6 mo, no progression at 12 mo	Not reported
Casale et al, 2022	Case report	1	MCC/left forearm	Multiple locoregional metastases	62	Intralesional T-VEC, total 4 doses (exact dose regimen not reported)		HSV	Pembrolizumab	Complete response	Mild fever, chills, aches
Nemunaitis et al, 2007	Clinical trial – open-label, single site phase 1, dose escalation	1 with BCC (Total 9 patients in trial)	BCC/Not mentioned (Trial included patients with various types of cancer)	Not mentioned	58	Intravenous ONYX-015 on d 1, 8, and 15 - Patient was in cohort 3, given 1 × 10^12^ viral particles per injection SC Etanercept 25 mg before, during and throughout cycle 1		Adenovirus	X Ray therapy, radiotherapy, xeloda, cisplatin	Stable disease	Transient grade 1 fever Other adverse events reported by study: Hyponatremia, dyspnea, pleural effusion, hypoalbuminemia, cerebral oedema, and anemia

*BCC*, Basal cell carcinoma; *HSV*, herpes simplex virus; *MCC*, Markel cell carcinoma; *T-VEC*, Talimogene laherparepvec.

∗ Study was primarily an animal model trial on melanoma skin cell lines, but included a case report in a patient with SCC, where OV was used as part of combination therapy.

† The 2 cases reported by this study continued follow-up and were reported again by Westbrook et al (2019). The duplicate cases were not included in quantitative analysis.

**Table II:** Published studies on the use of oncolytic virotherapy in cutaneous lymphoma treatment

Study	Study type	Number of patients	Diagnosis/location	Tumor stage/metastasis	Age	Treatment	Virus family	Previous treatment	Response	Adverse effects
Heinzerling et al, 2005	Clinical trial – open label, non-randomized, single center, phase 1	5	CTCL/variable locations: Cervical neck, plantar foot, upper arm, inguinal	Stage IIb and above	53-64	2 intratumoral injections of the measles virus (day 4 and day 17). Injections of IFN- (9 Mio U subcutaneous)were administered 72 h and 24 h before each of the measles-virus injections	Measles virus	Variable between patients – include surgical resection, RT, topical steroids, PUVA, methotrexate, acitretin, IFN-α	**Local response**: 1 patient with minor response, 3 with partial response, 1 with complete response **Response of distant lesions**: 2 patients with no response, 2 with partial response, 1 with complete response	Erythema at injection site, itching, stabbing sensation in left shoulder after injection, dizziness, arthralgia
Cook et al,∗ 2022	Clinical trial – single center, phase 1, dose escalation	4 with CTCL (Total 15 patients In trial)	CTCL/Only reported location for 2 patients – both had multiple lesions on limbs and trunk (Trial included patients with various types of relapsed refractory hematological malignancies (including CTCL, PTCL, MM, AML))	Not mentioned	33-88	Single intravenous infusion of VSV-IFNβ-NIS across 4 dose levels: - 5 × 10^9^TCID_50_ (*n* = 0) - 1.7 ×10^8^ TCID_50_ (*n* = 2) - 5 × 10^10^ TCID_50_ (*n* = 1) - 1.7 × 10^11^ TCID_50_ (*n* = 1)	Vesicular stomatitis virus	2-11 lines prior systemic chemotherapy	1 with partial response, 1 with progression of disease, 2 with stable disease that eventually progressed 2-3 mo post therapy	Hematologic adverse effects (eg cytopenias), fever, nausea, vomiting, hypotension, mild hypoxemia

*CTCL*, Cutaneous T cell lymphoma; *VSV*, vesicular stomatitis virus.

∗ Study is a subgroup of ongoing clinical trial NCT03017820 (summarized in Table IV).

Tumors had variable locations, involving the head and neck in 7 patients (35%), limbs in 6 patients (30%), trunk in 2 patients (10%), while 2 patients had multiple lesions on both trunk and limbs (10%). Location of tumors was not known for the remaining 3 patients. In terms of tumor stage, 6 patients (30%) had locoregional metastasis, 3 (15%) had nodal metastasis, and one (5%) had solid organ metastasis. Tumor stage and metastases were not specified for the remaining patients.

Among the identified studies, oncolytic viruses studied include Talimogene laherparepvec (T-VEC) (73%, *n* = 8), measles virus (9%, *n* = 1), vesicular stomatitis virus (9%, *n* = 1), and adenovirus (9%, *n* = 1).

Three case reports on OV in SCC treatment were included in our review. Two of the 3 cases (67%) reported the use of intralesional T-VEC monotherapy in relapsed/recurrent SCC, with complete response seen in both cases. The remaining case (33%, *n* = 1) reported combinational nivolumab with intralesional T-VEC therapy, with near-complete response of lesions seen.

Five studies on MCC were identified, all of which were case reports or case series that utilized intralesional T-VEC monotherapy. Of note, the 2 cases reported by Blackmon et al in 2017 continued follow up and were reported again by Westbrook et al with additional follow-up information. While both studies were included, only the results and follow-up information from Westbrook et al were taken into account in quantitative analysis. Of the total 7 patient cases reported, 85% (*n* = 6) showed complete response, while the remaining one patient had partial or near complete response.

Only one BCC case was identified. The patient was part of a clinical trial involving patients with various types of solid organ cancers, and received intravenous ONYX-015, an oncolytic virus from the adenovirus family. Stable disease was observed in this patient.

Less favorable responses were reported in patients with CTCL. Of the 5 CTCL patients treated with measles virus OV, only one (20%) demonstrated complete response, with the remaining 4 (80%) showing partial or minor response. Similarly, for the 4 CTCL patients treated with vesicular stomatitis virus (VSV) OV, one (25%) had partial response, one (25%) had progression of disease, and 2 (50%) had stable disease that eventually progressed. In addition, the study by Heinzerling et al also studied the effect of measles virus OV on distal lesions. However, similar results were seen, with complete response in only one patient (20%) and no response in 2 patients (40%). Notably, in CTCL, the OVs used differed from most other included studies – measles virus and VSV were used, compared to SCC and MCC, where studies all utilized T-VEC. The study by Cook et al also utilized intravenous administration rather than the conventional intralesional administration of most OVs.

### Adverse events

The most common reported adverse event was fever or flu-like symptoms, in 25% of reported cases (*n* = 5). Other reported adverse events include localized injection site reactions (10%, *n* = 2), fatigue (10%, *n* = 2), nausea or vomiting (10%, *n* = 2), and cytopenias (10%, *n* = 2). Other reactions such as hypotension (5%, *n* = 1), mild hypoxemia (5%, *n* = 1), arthralgia (5%, *n* = 1) and dizziness (5%, *n* = 1), were less common. In Lebhar et al, it was determined that the adverse events reported were unlikely related to OV treatment.

Nemunaitis et al had also reported several grade 3-4 adverse events, although it was not specified which of these were seen in the single BCC patient enrolled in the trial. Among the 9 patients enrolled, grade 3-4 adverse events seen include hyponatremia (*n* = 3, 33%), dyspnea (*n* = 2, 22%), pleural effusion (*n* = 1, 11%), hypoalbuminemia (*n* = 1, 11%), anemia (*n* = 1, 11%), dehydration (*n* = 1, 11%), and cerebral edema (*n* = 1, 11%). None of these adverse events were eventually attributed to OV treatment.

### Clinical trials

Fourteen unpublished phase 1 and 2 clinical trials were identified, of which 9 (64%) are ongoing, 2 (14%) are completed, and 3 (21%) were terminated or withdrawn (Tables III and IV).

**Table III:** Clinical trials on the use of oncolytic virotherapy in the treatment of nonmelanoma skin cancers and rare skin cancer subtypes

Trial	Study design	Phase	Status	Diagnosis	Virus family	Treatment regime	Primary outcome studied
NCT03773744 (Pelican)	Multicenter Open label	1b	Withdrawn (insufficient suitable drug supply)	Metastatic melanoma or cSCC that has failed prior standard of care treatments	Adenovirus (Ad-MAGEA3) Maraba virus (MG1-MAGEA3)	(1) CYC on D-3, IM Ad-MAGEA3 on D1, then IV MG1-MAGEA3 on D15, D18, pembrolizumab 200 mg (beginning either D1/wk 6) (2) IM Ad-MAGEA3 and Pembrolizumab on D1, IV MG1-MAGE3A on D15, IT MG1-MAGE3A on D22, 29, 36.	- Safety of administration in melanoma or cSCC - Determine maximum tolerated dose
NCT04349436 (ARTACUS)	Multicenter Open label	1b/2	Recruiting	Recurrent, locally advanced or metastatic cutaneous malignancies, including cSCC, BCC, MCC, and melanoma, where surgery or radiation is contraindicated	HSV	Intralesional RP1 every 2 wk	- Safety and tolerability - Efficacy
NCT01017185	Multicenter, open label, non-randomized	1	Completed	Refractory head and neck cancer, or solidtumors with cutaneous and/or superficial lesions (eg, cSCC, breast carcinoma, and malignantmelanoma)	HSV	Stage 1: Single intralesional HF10 (across different doses) Stage 2: Repeated intralesional HF10 at 1 × 10^6^ TCID50/dose and 1 × 10^7^ TCID50/dose	- Assessment of local tumor response
NCT04050436 (CERPASS)	Multicenter, randomized	2	Active, not recruiting	Metastatic or unresectable, locally advanced cSCC who are not candidates for surgery and/or radiation therapy	HSV	(1) IV Cemiplimab + intralesional RP1 every 3 wk (2) Cemiplimab monotherapy every 3 wk	- Objective response rate - Complete response rate
NCT04301011	Multicenter, open label, nonrandomized	1, 2A	Terminated (sponsor decision)	Patients with solid tumors Phase 2a section subdivided into 4 cohorts: (1) hepatic adenocarcinoma, (2) locally advanced or metastatic cutaneous melanoma, (3) locally advanced or metastatic cSCC, and (4) locally advanced or metastatic MSS-CRC	Vaccinia virus	(1) Intralesional Tbio-6517 ×4 doses ± booster up to 24 mo (2) Intralesional TBio-6517 ×4 doses ± booster up to 24 mo. IV Pembrolizumab from D9, every 3 wk up to 24 mo	- Incidence of adverse events in monotherapy and combination therapy - Maximum tolerated dose - Overall response rate
NCT04348916	Multicenter, open label	1	Terminated (due to sponsor portfolio reprioritization)	Advanced and/or refractory cutaneous, subcutaneous or metastatic nodal solid tumors or with liver metastases	HSV	(1) Intralesional ONCR-177 alone – dose escalation and dose expansion groups (2) Intralesional ONCR-177 + pembrolizumab – dose escalation and dose expansion groups	- Percentage of dose limiting toxicities - Percentage of adverse events - Percentage of serious adverse events - Maximum tolerated dose
NCT05076760 – Part 1	Nonrandomized, open label	1	Active, recruiting	Part 1 of study: Advanced/metastatic NSCLC, cSCC, MCC, melanoma, TNBC, pancreatic cancer, or head and neck cancer	Adenovirus	Part 1 of study: Intralesional MEM-288 (2 planned doses ± boosters up to maximum 6 doses, 3 weekly) at 3 dose cohort levels	- Maximum tolerated dose - Safety and tolerability (adverse events)
NCT04725331	Multicenter, open label, nonrandomized	1	Active, recruiting	Metastatic/advanced soft tissue sarcoma (STS), MCC, melanoma, triple negative breast cancer (TNBC) or nonsmall cell lung cancer (NSCLC)	Vaccinia Virus	Part A (dose escalation): repeated Intralesional BT-001 Part B: repeated intralesional BT-001 + IV pembrolizumab	- Safety and tolerability - Recommended dose - Overall response rate - Immune disease control rate at 6 mo
		2a				Phase 2A: expansion cohorts of repeated Intralesional BT-001 + IV pembrolizumab	
NCT03714828	Single center, open label, single group assignment	2	Completed	Low risk cSCC	HSV	Intralesional T-VEC ×4 injections	- Overall response rate
NCT03767348 (IGNYTE)	Multicenter, open label, nonrandomized	1	Active, recruiting	Advanced and/or refractory solid tumors	HSV	(A) Dose escalation of Intralesional RP1 in superficial and deep/visceral tumors (B) Dose expansion of Intralesional RP1 + IV nivolumab in superficial and deep/visceral tumors	- Percentage of adverse events - Percentage of serious adverse events - Percentage of dose limiting toxicities - Percentage of overall response rate - Maximum tolerated dose
		2		Various subgroups, including: - MSI-H or dMMR tumor – progressed on anti-PD1 treatment - NMSC including BCC, cSCC, basosquamous carcinoma, MCC – progressed on anti-PD1 treatment - Cutaneous melanoma, failed anti-PD1 treatment NSCLC, failed anti-PD1 treatment		Divided into subgroups based on diagnosis, each group given intralesional RP1 + IV nivolumab	
NCT05859074	Open label, nonrandomized	1	Recruiting	Advanced/relapsed/refractory solid tumors including cSCC, BCC, melanoma, MCC, sebaceous carcinoma, EMPD, Kaposi sarcoma, HNSCC, adnexal carcinoma, angiosarcoma, and cutaneous neoplasms that are separate primaries with morbidity from multiple surgeries that have failed standard therapy	Vaccinia virus	(1) Multidose monotherapy with intralesional MQ710 (2) Multidose intralesional MQ710 + IV pembrolizumab	Safety and tolerability
NCT04065152 (KAPVEC)	Multicenter, open label, single group assignment	2	Active, recruiting	Progressive Kaposi Sarcoma (KS) that does not require systemic therapy	HSV	Intralesional T-VEC (10^6^ pfu/ml at wk 1 then 10^8^/ml at wk 4 and every 2 wk for total 12 cycles – total duration 6 mo)	- Best overall response rate - Overall survival

*BCC*, Basal cell carcinoma; *HSV*, herpes simplex virus; *MCC*, Markel cell carcinoma; *T-VEC*, Talimogene laherparepvec.

**Table IV:** Clinical trials on the use of oncolytic virotherapy in the treatment of cutaneous lymphomas

Trial	Study design	Phase	Status	Diagnosis	Virus family	Treatment regime	Primary outcome studied
NCT03017820	Open label, nonrandomized	1	Active, recruiting	Multiple myeloma, acute myeloid leukemia, lymphomas, or histiocytic/dendritic cell neoplasms Includes cutaneous lymphomas and mycosis fungoides	Vesicular stomatitis virus	Group A: IV VSV-hIFNbeta-NIS alone Group B: IV VSV-hIFNbeta-NIS, ruxolitinib, cyclophosphamide Group C: VSV-hIFNbeta-NIS, ruxolitinib, nivolumab Group D: VSV-hIFNbeta-NIS, ruxolitinib, cemiplimab	• Incidence of adverse events
NCT05387226	Open label, single arm	1	Active, not recruiting	Relapsed T-cell lymphoma: CTCL, PTCL, AITL, ALCL		IV RT-01 (oncolytic virus injection) at wk 1 and 6, and every 8 wk thereafter	- Incidence of adverse events - Objective response rate - Disease control rate - Changes of immunoreactivity during treatment - Immunogenicity of RT-01 - Viral shedding - Maximum RNA peak concentration • Time of maximum RNA peak concentration

*CTCL*, Cutaneous T cell lymphoma; *VSV*, vesicular stomatitis virus.

Overall, they had significantly heterogeneous study designs investigating varying regimes, including OV as monotherapy (43%, *n* = 6), as combination therapy with existing immunotherapy (21%, *n* = 3), comparing combination versus monotherapy (29%, *n* = 4), or comparing combination therapy versus immune checkpoint inhibitor monotherapy (7%, *n* = 1). OVs from the herpes simplex virus family were the most studied (50%, *n* = 7). Other virus families studied include maraba virus and adenovirus combination therapy (*n* = 1), vaccinia virus (*n* = 3), adenovirus (*n* = 1), VSV (*n* = 1), and RP-01 oncolytic virus (*n* = 1). Studies also favored intralesional injection as the main method of administration (*n* = 11, 79%), compared to intravenous or intramuscular injections (*n* = 3, 21%).

## Discussion

In this study, we synthesized the data on using OV therapy in treating NMSCs, including CLs. Overall, among OVs studied, T-VEC has shown promising results in SCC and MCC, with minimal adverse events, while less favorable responses were seen in CLs. However, published data on OV therapy in rarer skin cancer subtypes, such as angiosarcoma, Kaposi sarcoma, and EMPD, is notably scarce. Among all viral vectors, herpes simplex virus viruses were the most studied in published studies and clinical trials. Clinical trials are ongoing to explore the role of several viral vectors as OVs and determine their optimal dose and regimen in treating various subtypes of NMSCs and CLs.

The ultimate goal of OV therapy is to develop an ideal vector that selectively infects tumor cells, self-replicates within them, and generates strong immune responses while minimizing collateral effects on normal cells. Therefore, understanding the structure and mechanisms by which OVs work is crucial to aid further enhancement of current available OVs.

### Mechanisms of action of OVs

OVs may be RNA or DNA viruses and can be classified into natural viral strains or genetically modified viruses.^7^ They are thought to mediate antitumoral activity via several mechanisms (Fig 1, *B*). First, they aim to cause direct tumor lysis by selectively infecting and replicating within cancer cells.^6^ OVs enter cells via recognition of specific receptors expressed on cells. Within cells, inherent abnormalities in cancer cell metabolism and replication support increased viral replication. OVs can also hijack cell protein synthesis, promoting viral product production.^8^ This triggers the lysis of infected cells.

Secondly, the lysis of infected cells can induce a systemic antitumor immune response, which helps to mediate tumor regression at distant tumor sites.^6^ The release of viral inflammatory signaling molecules, including neo-antigens, activates toll-like receptors and promotes the maturation of dendritic cells. This effect, in turn, activates antigen-specific CD4 and CD8 T cell responses. Activated CD8 cells can expand into cytotoxic effector cells with the ability to traffic to sites of established tumor growth, where they mediate anti-tumor immunity upon antigen recognition.^6^

Third, by remodeling the tumor microenvironment, OVs can counteract cancer-mediated immune invasion.^6^ Cancer cells typically avoid immune-mediated destruction by expressing surface receptors that inactivate immune cells and secrete factors that facilitate the recruitment of immune suppressive cells. However, OVs circumvent this via the release of viral particles that alter cytokines and immune cells within the tumor microenvironment to stimulate a local immune response. Lysis of tumor cells also releases neo-antigens that antigen presenting cells can take up and trigger a de novo immune response by T cells against the neo-antigen, even at distant sites.^6^^,^^8^

It is also postulated that some OVs can hinder tumor angiogenesis and facilitate tumor cell killing by restricting their supply of nutrients and oxygen. This occurs via inhibition of the synthesis of angiogenesis-related factors and OV infection of tumor vascular endothelial cells to cause cell lysis and cellular aggregation, which decreases tumor blood flow.^9^

### Challenges in OV development

Nevertheless, OV development has been plagued with several challenges. Firstly, ongoing research is being done to optimize the delivery of OV to target cells. While most OVs are administered intratumorally, this may not be feasible for all cancers, such as those presenting as multiple lesions over a large area.^9^^,^^10^ Although studies are ongoing to explore intravenous administration, they have faced significant unpredictability. For example, a host with a robust antiviral response may result in premature clearance of OVs and insufficient induction of antitumor immunity.^9^ This results in resistance to OV therapy with suboptimal bioavailability of OVs at tumor sites.^9^^,^^10^ To counteract this, several methods are being studied to prevent early viral neutralization, such as protective coatings for OVs with cell-derived nanovesicles, liposomes or chemical polymers, or by using cellular carriers.^9^ Other proposed strategies to enhance OV bioavailability include the concomitant administration of proteolytic enzymes (eg hyaluronidase or collagenase), which may contribute to extracellular matrix degradation, thereby enhancing OV spread within the tumor.^6^

Secondly, despite preliminary data showing that OVs are generally well tolerated,^10^ concerns remain over the safety of virotherapy. As the optimal dose at which OVs can achieve their desired effect with the least adverse effects is yet to be determined, there remains a need for high vigilance for potential adverse events. In fact, one of the most feared complications is the uncontrolled replication of OVs.^10^ A proposed “safety net” for such a situation is using virostatic compounds to halt viral replication. For example, herpes simplex virus-1 thymidine kinase can be used to halt host cancer cell and virus DNA replication in uncontrolled T-VEC replication.^10^

Furthermore, given viruses’ fast mutation rate and replicative potential, risks of viral shedding and unintentional transmission within the environment are additional considerations.^9^ This calls for standardized guidelines on the storage, preparation, and handling of OVs, as well as robust training and patient education on the management of OV injections.^9^

### Limitations

While OV development has made significant progress over the past decade, several considerations remain before it can be established as an anticancer therapy in NMSC and CL. Our review is limited by the relative paucity of published studies on OV in NMSC and CL as compared to melanoma, and as current data are largely limited to case reports and few trials, it remains unclear whether such responses can translate into significant clinical benefit in larger scale clinical trials and in practice. Furthermore, current clinical trials vary considerably in terms of study populations, design, and response assessment, making generalization of results difficult. Although preliminary data are promising, OVs still have a long way to go before they cement their role in skin cancer treatment.

## Conclusion

Oncolytic viruses are an emerging anticancer therapy. While current data on OV use in NMSC treatment are encouraging, there is a lack of solid phase III clinical trial evidence to support these findings, while current trials are limited by their significant heterogeneity. Further studies would aid our understanding of the potential role of OVs in shaping the future of NMSC and CL treatment.

## Conflicts of interest

None disclosed.
